# Impact of vancomycin resistance on mortality in neutropenic patients with enterococcal bloodstream infection: a retrospective study

**DOI:** 10.1186/1471-2334-13-504

**Published:** 2013-10-29

**Authors:** Sung-Yeon Cho, Dong-Gun Lee, Su-Mi Choi, Jae-Cheol Kwon, Si-Hyun Kim, Jae-Ki Choi, Sun Hee Park, Yeon-Joon Park, Jung-Hyun Choi, Jin-Hong Yoo

**Affiliations:** 1 Division of Infectious Diseases, Department of Internal Medicine, Vaccine Bio Research Institute, College of Medicine, The Catholic University of Korea, Seoul, Republic of Korea; 2 The Catholic Blood and Marrow Transplantation Center, Seoul St. Mary’s Hospital, College of Medicine, The Catholic University of Korea, Seoul, Republic of Korea; 3 Department of Internal Medicine, Daniel Hospital, GyeongGi-Do, Republic of Korea; 4 Department of Life Sciences, Pohang University of Science and Technology, Pohang, Republic of Korea; 5 Department of Laboratory Medicine, College of Medicine, The Catholic University of Korea, Seoul, Republic of Korea

**Keywords:** Bacteremia, Enterococcus, Mortality, Neutropenia, Risk factors, Vancomycin resistance

## Abstract

**Background:**

Vancomycin-resistant *Enterococcus* (VRE) bloodstream infection (BSI) is generally associated with the delayed administration of adequate antibiotics. The identification of risk factors and outcomes of VRE BSI is necessary for establishing strategies for managing neutropenic fever in patients with hematological malignancies.

**Methods:**

We retrospectively analysed consecutive cases of enterococcal BSI in patients with neutropenia after chemotherapy or stem cell transplantation between July 2009 and December 2011 at a single center.

**Results:**

During the 30-month period, among 1,587 neutropenic patients, the incidence rate of enterococcal BSI was 1.76 cases per 1,000 person-days. Of the 91 enterococcal BSIs, there were 24 cases of VRE. VRE BSI was associated with *E. faecium* infection (*P* < .001), prolonged hospitalization (*P* = .025) and delayed administration (≥48 hours after the febrile episode) of adequate antibiotics (*P* = .002). The attributable mortality was 17% and 9% for VRE and vancomycin-susceptible *Enterococcus* (VSE), respectively (*P* = .447). The 30-day crude mortality was 27% and 23% for VRE and VSE, respectively (OR 1.38, 95% CI 0.53–3.59; *P* = .059). Only SAPS-II was an independent predictive factor for death (adjusted OR 1.12, 95% CI 1.08–1.17; *P* < .001).

**Conclusions:**

In conclusion, vancomycin resistance showed some trend towards increasing 30-day mortality, but is not statistically significant despite the delayed use of adequate antibiotics (≥48 hours). Only underlying severity of medical condition predicts poor outcome in a relatively homogeneous group of neutropenic patients.

## Background

Enterococcal bloodstream infections (BSI) have been increasing in hospitalized patients since the 1990s [1,2]. *Enterococci* are normal intestinal flora of humans, but usually cause infections when immunity of the host is compromised [3]. At our Blood and Marrow Transplantation Center, *Enterococcus* has emerged as the third most common pathogen, constituting 14.0% of all BSIs, and vancomycin resistant *Enterococcus* (VRE) was responsible for 20.6% of proven enterococcal BSIs [4]. There are several studies on outcomes after administration of newer antimicrobial agents such as linezolid, daptomycin, quinupristin-dalfopristin or tigecycline [5,6]. But VRE BSI is still a major concern, because of the limited therapeutic options available and higher mortality subsequent to infection [7,8].

It has been demonstrated that vancomycin resistance increases mortality in patients with enterococcal BSI [1,9-14]; however, controversy remains [5,15-19]. Conflicting results on the clinical implications of vancomycin resistance may have been due to the varied severity of the underlying illness in patients with VRE BSI. Previous meta-analysis concluded that vancomycin resistance is independently associated with increased mortality in enterococcal BSI [1]. However, only a subset of the studies involved in the meta-analysis examined immunocompromised individuals [13,14].

Whether vancomycin resistance increases mortality in patients with enterococcal BSI could be one of the important considerations in determining empirical therapeutic approach, especially in the setting of chemotherapy-induced reversible neutropenia. This study examined the risk factors and outcomes of VRE BSI as compared with vancomycin-susceptible *Enterococcus* (VSE) BSI, and the factors associated with mortality in a relatively homogeneous group of patients with neutropenia after chemotherapy or stem cell transplantation (SCT) for underlying hematologic diseases.

## Methods

### Study design and clinical setting

We retrospectively reviewed all consecutive episodes of enterococcal BSIs in adult patients with neutropenic fever from July 2009 to December 2011 at the Catholic Blood and Marrow Transplantation Center, Seoul St. Mary’s Hospital. Our hospital is a 1,200-bed, university-affiliated, tertiary-care center in Seoul, South Korea. The Catholic Blood and Marrow Transplantation Center performs over 350 SCTs annually.

Eligible patients included those with hematological malignancies, who experienced neutropenic fever during chemotherapy or SCT, who were older than 18 years of age, and were documented to be blood culture positive for *Enterococcus* species. Enterococcal BSI cases were obtained from a computerized microbiology database. We collected data targeted to positive blood culture results from chemotherapy ward and SCT ward. In cases with persistent BSI, we analysed the first episode. If cases met the definition of separate BSI, each infection was considered individually. Concomitant VRE and VSE BSI were excluded. Even in cases with neutropenic fever and documented enterococcal BSI, patients who had not previously received treatment for hematological malignancies, such as anti-cancer chemotherapy or SCT, were excluded.

The data obtained for each patient included age, sex, underlying diseases, absolute neutrophil count (ANC) at the onset of fever, severity and duration of neutropenia, severity of illness (Simplified Acute Physiology Score, SAPS II) and Charlson’s comorbidity index at the onset of BSI, length of hospital stay, stay in intensive care unit (ICU) before the onset of BSI, septic shock, the presence of previously documented rectal VRE colonization, organisms isolated from blood and antimicrobial susceptibility, antibiotics administered, and survival 7 and 30 days after the onset of BSI.

Blood for cultures was sampled using sterile techniques, with one set from peripheral vein puncture and another set simultaneously from a central venous catheter. Each 10~15 mL of blood was inoculated into aerobic and anaerobic bottles (BD BACTEC™ Plus Aerobic/F, Lytic/10 Anaerobic/F Culture Vials, Becton Dickinson, Sparks, MD, USA), which were immediately transported to the clinical laboratory. Antibiotic susceptibility testing was performed with an automatic system (Vitek-2, bioMérieux, Hazelwood, MO, USA). *E*. *faecalis* clinical isolates are almost always susceptible to ampicillin; hence, ampicillin sensitivity of *E. faecalis* was not tested in our study. Polymerase chain reaction (PCR) test to look for van A, B, C genes was not performed routinely.

At our center, during the chemotherapy or SCT, oral ciprofloxacin (500 mg twice a day) is used as routine prophylaxis. The initial empirical treatment of neutropenic fever in hematological malignancy patients is to use anti-pseudomonal cephalosporin (cefepime or ceftazidime) and/or an aminoglycoside (isepamicin), excluding the initial use of glycopeptides. The use of glycopeptides as empirical antimicrobial therapy is recommended if the patient’s blood cultures are positive for Gram-positive bacteria, a catheter-related infection is suspected, there is a history of MRSA infection, the patient has severe sepsis or shock pending the results of cultures, the patient has severe mucositis, or the patient has skin or soft tissue infection. If Gram-positive bacteremia persists for 2 or 3 days after adding glycopeptides, we consider anti-VRE therapy before receiving the final microbiology results [20,21].

Rectal VRE surveillance is not routinely performed in patients undergoing chemotherapy or SCT according to ASBMT guidelines [22]. For cases of previous VRE infection or hospitalization in the ICU, rectal swabs for VRE surveillance are performed. All VRE-colonized or -infected persons are placed under contact precautions for nosocomial infection control until they have three consecutive negative results.

The Institutional Review Board of Seoul St. Mary’s hospital approved the research protocol with a waiver of informed consent (KC12RISI0314).

### Definitions

Enterococcal BSI was defined as isolation of *Enterococcus* species in two or more blood culture results [1,8,13,15,17]. Enterococcal BSI occurring 60 or more days after a previous initial episode was considered a separate episode [8,12]. Neutropenia was defined as an ANC <500/mm^3^, or <1000/mm^3^ with predicted falls to <500/mm^3^ within 2~3 days. Severe neutropenia was defined as an ANC <100/mm^3^[20,23]. Fever was defined as an increase in body temperature to over 38.0°C using a tympanic thermometer or to over 37.5°C using an axillary thermometer [20]. The length of hospitalization was defined as the number of days from hospital admission to the development of clinically significant enterococcal bacteremia. Intermediate susceptibility was classified as 'non-susceptible’. Clinical outcomes were classified as 'improvement’ indicating resolution of the signs of systemic inflammatory response syndrome (SIRS), 'failure’ for any sustained signs of infection or death [24]. Microbiologic outcomes were classified as 'eradication’ or 'persistence’ [11]. When any follow-up blood culture result was positive for *Enterococcus* species, the case was considered as 'persistence’. When 7-day microbiologic outcome showed 'persistence’, it considered as 'microbiologic failure’ at 7 day after the febrile episode. Mortality was considered attributable to the *Enterococcus* if the patient died within 7 days of the BSI and no other cause could be identified. Crude mortality was defined as mortality that occurred within the month that followed a BSI episode [25]. Empirical antimicrobial therapy was considered to be appropriate when at least one active antimicrobial agent susceptible to the organism by in vitro test was administered [26]. The length of delay until adequate antibiotics treatment was defined as the interval between the onset of fever and the time that antibiotics to which the organism was susceptible were administered. Delayed administration of adequate antibiotics was defined when the length of the delay was 48 hours or more [12].

### Statistical analysis

To identify factors associated with the development of VRE BSI, chi-square analysis or Fisher’s exact test was used to compare categorical variables, and a Student’s *t*-test or Mann–Whitney U test was used to compare continuous variables. In order to identify factors associated with mortality, Cox proportional hazard model with backward method was used to control for the effects of confounding variables. Kaplan-Meier survival curves were used to analyse the mortality trends, and the results were compared using the log-rank test. SAPS-II score at the onset of febrile episode was analysed by using receiver operating characteristic (ROC) curve. For measures of association, a 2-tailed *P* < .05 was considered to be significant. Statistical analysis were performed using SAS software ver. 8.2 (SAS Institute Inc., Cary, NC, USA).

## Results

During the 30-month period, among a total of 2,028 patients admitted with hematological malignancies, 1,587 developed neutropenia during chemotherapy or SCT, and 91 episodes of enterococcal BSIs were identified. Of these infectious episodes, 24 (26.4%) were VRE BSIs. The incidence rate of enterococcal BSI in neutropenic patients who underwent chemotherapy or SCT was 1.76 cases per 1,000 person-days. There were 2 cases of concurrent VRE and VSE bacteremia. It was excluded because of the interpretative problem. A review of monthly incidence rates of enterococcal BSIs showed that no outbreaks occurred during this study period.

The baseline characteristics are shown in Table 1. There were no differences in age, sex, SAPS-II and Charlson’s comorbidity index, and the duration and severity of neutropenia between VSE and VRE BSI groups. VRE BSI was associated with *E. faecium* infection (*P* < .001) and prolonged hospitalization (*P* = .025). All of the patients in this study had a central venous catheter for their chemotherapy or SCT, which was Hickman catheter or chemoport. Delayed administration of adequate antibiotics (more than 48 hours after the febrile episode) was more common in VRE BSI than in the VSE BSI group (*P* = .002). The median interval from the onset of the febrile episode to the administration of adequate antibiotics was 1.4 (range <1 ~ 5) days in the VSE BSI group and 2.8 (range <1 ~ 5) days in the VRE BSI group. Enterococcal BSI developed a median of 14 days after the onset of neutropenia. In the VSE BSI group, 61.2% of the patients had acute myeloid leukaemia (AML), while acute lymphoblastic leukaemia (ALL) was the major underlying disease (54.2%) in the VRE BSI group (*P* = .046).

**Table 1:** Descriptive baseline characteristics of patients with enterococcal bloodstream infections

	**VSE BSI (n=67)**	**VRE BSI (n=24)**	***p-value***
Age, years	48.0±14.0	44.9±14.5	0.356
Male sex	41 (61.2)	10 (41.7)	0.098
Underlying hematologic diseases			**0.046**
AML	41 (61.2)	10 (41.7)	0.098
ALL	18 (26.9)	13 (54.2)	**0.015**
Others^a^	8 (11.9)	1 (4.2)	0.436
Treatment for underlying diseases			0.861
Chemotherapy	56 (83.6)	20 (83.3)	>0.999
Allogeneic SCT	9 (13.4)	4 (16.7)	0.738
Autologous SCT	2 (3.0)	0 (0)	>0.999
Enterococcal species			**<0.001**
*E. faecium*	44 (65.7)	22 (91.7)	**0.016**
*E. faecalis*	23 (34.3)	0 (0.0)	**<0.001**
*E. gallinarum*	0 (0.0)	2 (8.3)	0.067
SAPS II at the onset of BSI	39 (28–95)	41 (31–74)	0.336
Charlson’s comorbidity index	2 (0–4)	2 (0–4)	0.644
ICU care	16 (23.9)	7 (29.2)	0.609
Septic shock	8 (11.9)	2 (8.3)	>0.999
Length of hospitalization, day	22 (13–44)	26 (17–77)	**0.025**
Duration of neutropenia before BSI, day	14 (0–156)	15 (6–27)	0.908
Severity of neutropenia			0.112
ANC <500/mm^3^	2 (3.0)	3 (12.5)	
ANC <100/mm^3^	65 (97.0)	21 (87.5)	
Administration of adequate antibiotics			**0.002**
≤48 hours after febrile episode	50 (75.8)	10 (41.7)	
>48 hours after febrile episode	16 (24.2)	14 (58.3)	

Data are presented as n (%), mean±SD or median (range). *Abbreviations*: *ALL* acute lymphoblastic leukemia, *AML* acute myeloid leukemia, *ANC* absolute neutrophil count, *BSI* bloodstream infection, *ICU* intensive care unit, *SAPS II* Simplified Acute Physiology Score, *SCT* stem cell transplantation, *VRE*, vancomycin-resistant enterococci, *VSE* vancomycin-susceptible enterococci. ^a^Myelodysplastic syndrome (n=2), multiple myeloma (n=1), aplastic anemia (n=3), lymphoma (n=2) and hemophagocytic lymphohistiocytosis (n=1).

**Figure 1:**
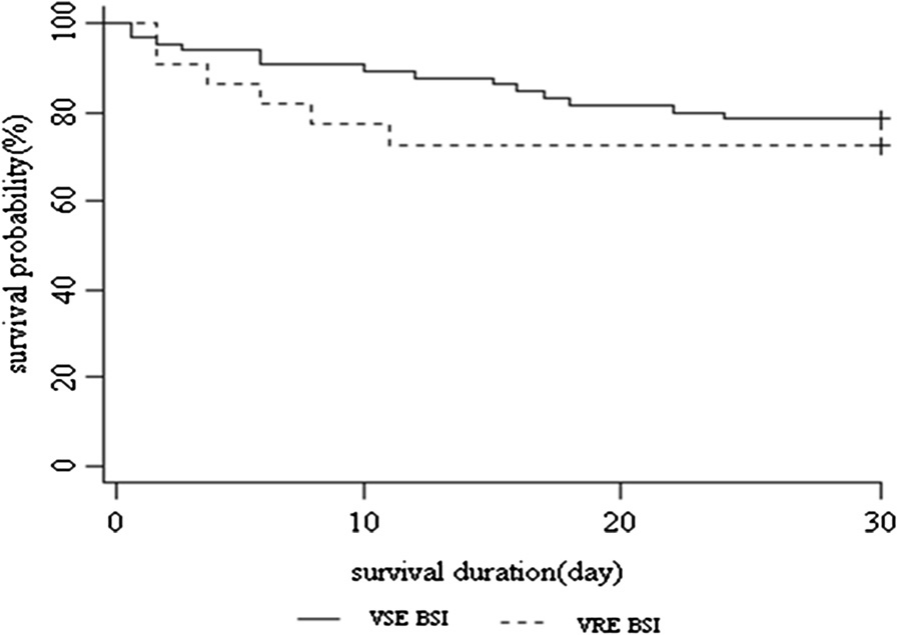
**Kaplan-Meier curves for survival.** Kaplan–Meier curves for survival during episodes of bloodstream infections (BSIs) with vancomycin-resistant enterococci (VRE) versus vancomycin-susceptible enterococci (VSE) (Log rank test, *P* = .509).

The outcomes are shown in Table 2. The 7-day clinical treatment failure rate was 50.0% and 26.9% in VRE and VSE BSI, respectively (*P* = .040), while microbiologic failure at 7 days after the onset of BSI did not differ significantly (16.7% and 11.9%, *P* = .499). Despite the delayed administration of adequate antibiotics and delayed resolution of the signs of SIRS in the VRE BSI group, the attributable mortality was 16.7% for VRE and 9.0% for VSE BSI (*P* = .447). The 30-day crude mortality was 27.3% for VRE and 22.7% for VSE (hazard ratio [HR] 1.38, 95% confidence interval [CI] 0.53–3.59; *P* = .059). Kaplan–Meier curves comparing patient survival are shown in Figure 1. In the Cox proportional hazard analysis, with the satisfaction of the assumption of 'proportional hazards’, only SAPS-II at the onset of BSI proved to be an independent risk factor for death (adjusted HR 1.12, 95% CI 1.08–1.17; *P* < .001) (Table 3). Analysing by ROC curve, SAPS-II at the onset of febrile episode is a good predictive factor for 30-day crude mortality with area under the curve (AUC) value of 0.817 (*P* <.001). With a cut-off value of 42.5, the sensitivity and specificity for 30-day crude mortality was 0.810 and 0.791, respectively.

**Table 2:** Outcomes of enterococcal bloodstream infection in the VSE and VRE BSI groups

	**Total (n=91)**	**VSE BSI (n=67)**	**VRE BSI (n=24)**	***P-value***
7-day clinical treatment failure	30 (33.0)	18 (26.9)	12 (50.0)	**0.040**
7-day microbiologic failure	12 (13.2)	8 (11.9)	4 (16.7)	0.499
Mortality				
Attributable mortality	10 (11.0)	6 (9.0)	4 (16.7)	0.447
Crude mortality	21 (23.9)	15 (22.7)	6 (27.3)	0.059

Data are presented as n (%). *Abbreviations*: *BSI* bloodstream infection, *VRE* vancomycin-resistant *enterococci*, *VSE* vancomycin-susceptible *enterococci*.

**Table 3:** Factors associated with 30-day mortality in patients with enterococcal bloodstream infections

	**Unadjusted HR (95% CI)**	**Adjusted HR (95% CI)**
Age, years	1.03 (0.99–1.06)	
Male sex	1.00 (0.62–1.62)	
Vancomycin resistance	1.38 (0.53–3.59)	0.75 (0.24–2.36)
SAPS II at the onset of BSI (per 1 point)	**1.12 (1.08–1.17)**	**1.13 (1.08–1.18)**
Underlying hematologic diseases		
AML	1.00	
ALL	0.78 (0.27–2.24)	
Others	2.29 (0.73–7.19)	
Treatment for underlying diseases		
Chemotherapy	1.00	
SCT	1.33 (0.72–2.47)	
Length of hospitalization, day	**1.05 (1.02–1.09)**	1.00 (0.98–1.02)
Duration of neutropenia before BSI, day	1.00 (0.98–1.02)	
Severity of neutropenia		
ANC (<500/mm^3^)	1.00	
ANC (<100/mm^3^)	0.43 (0.10–1.86)	
Delayed administration of adequate antibiotics (> 48 hours)	0.78 (0.30–2.04)	

*Abbreviations*: *ALL* acute lymphoblastic leukemia, *AML* acute myeloid leukemia, *ANC* absolute neutrophil count, *BSI* bloodstream infection, *CI* Confidence interval, *HR* Hazard ratio, *ICU* intensive care unit, *SAPS II* Simplified Acute Physiology Score, *SCT* stem cell transplantation, *OR* Odds ratio, *VRE* vancomycin-resistant enterococci, *VSE* vancomycin-susceptible enterococci.

Table 4 shows the susceptibility patterns according to *Enterococcus* species. Of the 91 isolates, 66 (72.5%) were *E*. *faecium,* 23 (25.3%) were *E*. *faecalis*, and 2 (2.2%) were *E*. *gallinarum*. One-third of the *E*. *faecium* isolates (22 of 66) were resistant to vancomycin. Both *E*. *gallinarum* isolates were resistant to vancomycin. Of the *E*. *faecium* isolates, all were resistant to ampicillin, 75.8% (50 of 66) showed high-level resistance to gentamicin, and all were susceptible to quinupristin-dalfopristin. Two *E*. *faecium* isolates were not susceptible to linezolid: one isolate showed intermediate susceptibility and one showed resistance; in both, the MIC changed to susceptible when the blood cultures were repeated. Among the *E*. *faecalis* isolates, 69.6% (16 of 23) were resistant to high-level gentamicin, and all isolates were susceptible to glycopeptides and linezolid. For tigecycline, 97.0% (64 of 66) of the *E*. *faecium* and 95.7% (22 of 23) of the *E*. *faecalis* isolates were susceptible.

**Table 4:** **In vitro susceptible rate according to *Enterococcus *** **species**

	***E. faecium *** **(n=66)**	***E. faecalis *** **(n=23)**	***E. gallinarum *** **(n=2)**
Ampicillin	0 (0)	**-**	2 (100)
High level gentamicin synergy	16 (24.24)	7 (30.43)	2 (100)
Vancomycin	44 (66.67)	23 (100)	0 (0)
Teicoplanin	47 (71.21)	23 (100)	2 (100)
Linezolid	64 (96.97)	23 (100)	2 (100)
Quinupristin-dalfopristin	66 (100)	0 (0)	0 (0)
Tigecycline	64 (98.46)	22 (95.65)	2(100)

Data are presented as n (%).

Among the 24 VRE BSI patients, rectal swabs for VRE were performed in 20 patients, 90% (18 of 20) of whom were positive. Results of rectal swabs done previous to diagnosis of VRE BSI were only available in 9 patients, 2 of whom (22.2%) showed a positive result, indicating rectal VRE colonization prior to BSI.

We conducted a subgroup analysis of the 66 *E*. *faecium* BSIs to eliminate the differences that may have resulted from inclusion of multiple enterococcal species in the overall study cohort. This analysis showed concordant mortality results between vancomycin-susceptible and vancomycin-resistant *E*. *faecium*.

## Discussion

This study examined the impact of vancomycin resistance on mortality in neutropenic patients with enterococcal BSI. The results in this study indicate that vancomycin resistance does not affect the 7-day and 30-day mortalities in a relatively homogeneous group of patients. A meta-analysis estimated that VRE BSI is associated with a 2.52-fold increased mortality [1]. However, it is still controversial whether VRE BSI results in higher mortality than VSE BSI in neutropenic patients [12,18,19]. In general, the clinical impact of vancomycin resistance is thought to be more pronounced in immunocompromised patients. Several reports indicate that hematological malignancy is a risk factor for VRE infections [7,8]. However, the use of antibiotics and severity of underlying disease should be considered in any outcome analysis because outcomes may be affected by variables such as antibiotic treatment strategies during treatment for neutropenic fever.

There was a significant delay in effective anti-VRE therapy for over 48 hours, and the maximum delay was 5 days based on the culture results in the VRE BSI group. However, despite the delayed administration of adequate antibiotics and delayed improvement of clinical signs of SIRS in the VRE group, the 7- and 30-day mortality rates did not differ between the two groups. One report indicates that VRE is a dominant pathogen before engraftment in the setting of SCT, emphasizing the potential utility of the empirical treatment of Gram-positive organisms in the pre-engraftment period in febrile SCT patients [14]. But, there are many considerations to decide empirical antibiotics including incidence, risk and outcomes. In addition, virulence of the pathogen, breakthrough infection, overtreatment and emerging resistance are also important in decision making. Our results suggest that clinicians need to be careful to add anti-VRE therapy empirically or pre-emptively before obtaining the final microbiology results. Current antibiotics strategy of adding glycopeptides in febrile neutropenic patients with presumptive Gram-positive bacteremia might be sufficient on the premise that treatment must be individualized.

Mortality related to VRE BSI has been reported to vary from 4~80% [9-18,27,28]. As mentioned above, such a broad range may stem from the diverse underlying conditions and different antimicrobial strategies. Recently, Vydra *et al*. reported that mortality 30 days after enterococcal infection was 38% for both VRE and VSE BSI cases in SCT patients and non-relapse mortality 1 year after transplant was 53% and 33% for VRE and VSE BSI cases respectively [27]. These results are similar to ours, as the 30-day mortality showed no difference between the two groups, while the 1-year mortality after SCT in VRE infections may reflect the severity of the underlying medical condition, rather than the poor outcome portended by vancomycin resistance itself. Interestingly, higher vancomycin resistance rate was reported in their cohort, with 66% VRE BSI among enterococcal BSI compared to ours. In their cohort, standard antibiotic therapy included ceftazidime, vancomycin, and often tobramycin until results of cultures were obtained, and such a strategy may have influenced the vancomycin resistance rate in enterococcal BSIs.

Possible causes that our results showed no difference between the VSE and VRE BSI group include the following; first, the known higher mortality in the VRE BSI group may be due to host factors, rather than a pathogen-related factor [5,19]. Our study population was a relatively homogeneous group that showed neutropenia after treatment for hematological malignancies. The statistically equivalent initial SAPS II at the onset of BSI may have contributed to the similar outcome of the two groups. Second, the differences in virulence amongst the enterococcal species can be considered. *E*. *faecium* is more frequently associated with vancomycin resistance than is *E*. *faecalis*. Some studies have examined animal models, with one study using an intestinal model of *Caenorhabditis elegans* reporting that low inoculums of *E*. *faecalis* grow to a high titer in the *C*. *elegans* intestine, resulting in persistent infection and death, whereas a high ingested titer of *E*. *faecium* also accumulates in the nematode gut, but did not cause death [29]. Another animal study reported that in an intraperitoneal mouse model, a greater inoculum size was required with *E*. *faecium* than with *E*. *faecalis* strains [30]. Based on these results, the higher virulence of *E*. *faecalis* species may have impacted upon the similar mortality shown in the VSE and VRE groups.

Interestingly, a difference in the vancomycin resistance rate between myeloid and lymphoid malignancy was observed, with more patients with ALL in the VRE group. There are some reports that neutrophils play a critical role in the pathogenesis of enterococcal infections [31,32]. However, the immune responses to *Enterococci* are poorly understood. Our finding is not consistent with the results of Worth *et al*., who identified an underlying diagnosis of AML as a risk factor for VRE [33]. Theirs was a case–control study between patients with and without VRE infection, not between VRE- and VSE-infected groups. Moreover, there was an outbreak of VRE infection in the hematology unit, and empirical antibiotic therapy was modified during the study period. These factors may explain discrepancies found between their results and ours, although further studies are needed.

According to CDC criteria for nosocomial infection, enterococcal BSI was defined as any blood culture growing *enterococcus* species [34]. But diverse definitions have been used for each study (≥1 or 2 blood culture results), which may result in selection bias. Therefore, to use a strict BSI definition, we included cases with two or more positive blood cultures of enterococcal species [1,8]. We also included only BSI cases that occurred during neutropenia related to their treatment with exclusion of cases undergone palliative care. This study design made our study population as a relatively homogeneous group with reversible neutropenia. And we calculated the incidence rate of enterococcal BSI considering hospital stay. In addition, a subgroup analysis was performed for *E*. *faecium* BSI, and gave concordant results. Our investigations have important implications for clinical practice.

Nevertheless, our study has some limitations. First, it was a retrospective study. The retrospective design made it difficult to evaluate the duration of bacteremia and gastrointestinal disease. Second, VRE surveillance is not routinely performed in our center, data for VRE colonization would be insufficient [22,35]. Third, we selected the definition of enterococcal BSI with 2 or more blood culture positivity, the incidence rate might be affected. Fourth, it had a limitation stemming from its sample size. Based on a previous meta-analysis [1], we assumed a crude mortality for the VSE BSI group of 20% and an odds ratio (OR) for vancomycin resistance on mortality of 2.52. Based on these assumptions, the statistical power of this study to detect a significant difference in mortality is a power of 40% in a 2-sided test with a probability of 0.05. However the mortality data and the OR to calculate the sample sizes are not for an immunocompromised population and may include confounding effects of underlying diseases and severity.

## Conclusions

In summary, our data suggest that enterococcal BSI due to vancomycin-resistant species in patients with hematological malignancies tends to occur after a long period of hospitalization. SAPS-II predicts poor outcome, reflecting patient age and the severity of illness. By contrast, severe neutropenia and vancomycin resistance itself did not appear to affect the fatality rate. The attributable and crude mortality were not different between VRE and VSE BSI groups despite the delayed use of adequate antibiotics in a relatively homogeneous immunocompromised group. Further prospective controlled studies are needed.

## Competing interests

The authors declare that they have no competing interests.

## Authors’ contributions

SYC conducted the hospital chart review and analysis of resulting data, interpreted data, drafted the initial study report and wrote the final report. DGL conceptualized the study and formulated the study design, and contributed to data interpretation. He revised and edited the manuscript. SMC participated in critical revision of manuscript. JCK, SHK, SHP, JHC and JHY contributed the data interpretation and editing the manuscript. JKC participated in data collection and study logistics. YJP contributed the interpretation of microbiologic data. All authors read and approved the final manuscript.

## Pre-publication history

The pre-publication history for this paper can be accessed here:

http://www.biomedcentral.com/1471-2334/13/504/prepub

## Previous presentation

Part of this study was presented at the 52^nd^ Interscience Conference on Antimicrobial Agents and Chemotherapy in San Francisco, September 9–12, 2012 (K-1632).
